# The Use of Adjuvant Dexamethasone in Chronic Subdural Hematoma After Surgery

**DOI:** 10.7759/cureus.44086

**Published:** 2023-08-25

**Authors:** Hidayet Safak Cine

**Affiliations:** 1 Neurosurgery, Istanbul Medeniyet University, Prof. Dr. Suleyman Yalcin City Hospital, Istanbul, TUR

**Keywords:** radiological membrane, subdural dimensions, glucocorticoids, dexamethasone, subdural hematoma

## Abstract

Objective: The use of oral anticoagulants in elderly patients is a predisposing factor in developing a subdural hematoma (SDH). The surgical option is often the standard approach but can be accompanied by complications. In this study, we hypothesized that dexamethasone administration after surgery would positively affect subdural change and subdural size in the second week.

Materials and methods: Within the scope of this prospective research, 66 individuals who underwent surgical intervention for chronic subdural hematoma (CSH) at the neurosurgery department either as an outpatient or under emergency circumstances have been enrolled in the study. During the examination, the patients were questioned about the traumatic incident, the localization of injury, and the utilization of anticoagulant and antiplatelet medications. The Glasgow Coma Scale (GCS) was administered to all patients to assess neurological deficits. Computed tomography (CT) was utilized to determine the characteristics of the hematoma.

Results: A total of 66 patients, 22 (33.3%) using and 44 (66.6%) not using dexamethasone, were included in the study. Analysis of variance (ANOVA) indicated that a statistical difference was achieved in the second week after the operation (p<0.043). During the examination of subdural dimensions after the operation and in the second postoperative week, a difference was detected between the radiological membrane and subdural dimensions. A statistically significant correlation was found between anticoagulation and the type of hematoma (p<0.025).

Conclusion: Regarding the outcomes of this research, we can conclude that dexamethasone was not associated with any adverse events. Additionally, dexamethasone could leverage reoperation prevention for the elderly with various comorbidities.

## Introduction

Chronic subdural hematoma (CSH) is a condition that is commonly observed in the geriatric population. The accumulation of fluid from hemorrhage within the subdural cavity is the definition of the condition. The prevalence of this condition is relatively elevated, with an average range of 1-2 cases per 100,000 individuals. CSH is a common consequence of a mild traumatic brain injury. According to a study [1], head trauma can occur with a minimal nature that patients may not be aware of the exact moment of trauma. Typically, individuals seek admission to the medical facility with clinical presentations that are relatively mild. Gradual cerebral compression can lead to the gradual onset of headaches and progressive neurological impairments or balance disturbances. Occasionally, elderly patients who have been neglected may present to the hospital with a state of impaired consciousness. Individuals who have neurological impairment are typically treated with surgery. Also, the dimensions of the hematoma are also used in the decision of surgery [2].

The use of anticoagulants in geriatric patients is commonly regarded as a risk factor for the onset of CSH. Because of the old age of patients, the surgery is also accompanied by potential complications. A significant proportion of patients necessitate subsequent surgical interventions. Consequently, there is a possibility of an escalation in morbidity and mortality rates, necessitating medical interventions to avert the need for recurrent surgical procedures [3].

Apart from leakage bleeding, CSH is commonly attributed to the prolonged inflammatory response as its primary etiology. Following the inflammatory response, a complex series of events takes place, involving the formation of new membranes, hyperfibrinolysis, and angiogenesis [4]. Exudation fluid can also accumulate. The possibility of rebleeding within the exudate fluid exists, which may manifest CSH in a subacute form. For this purpose, glucocorticoids have been used in many diseases to reduce the recurrence of CSH [5-7].

CSHs may result in long-term complications, including rebleeding and leakage [5]. Surgical intervention is only deemed necessary when the hematoma exceeds a size of 1 cm [6]. Simultaneously, despite the implementation of a surgical drain, there exists the possibility of fluid accumulation and subsequent elevation of subdural distance over a period of time [7]. The present investigation hypothesized that the administration of dexamethasone subsequent to surgical intervention would yield a favorable impact on subdural alterations and dimensions after the second week of operation. The administration of adjuvant dexamethasone during the postoperative phase may potentially reduce the need for reoperation in patients who are elderly and have comorbidities.

## Materials and methods

In this prospective research, 66 individuals who underwent surgical intervention for chronic subdural hematoma were enrolled, whether treated as outpatients or under emergency circumstances. The patients were divided into two groups: the dexamethasone group and the control group. All procedures followed were in accordance with the ethical standards of the responsible committee on human experimentation (institutional and national) and with the Helsinki Declaration of 1975, as revised in 2008. Ethics committee approval has been granted from our institution, and informed consent has been obtained from all participants.

During the examination, the patients were questioned about the traumatic incident, the localization of injury, and the utilization of anticoagulant and antiaggregant medications. The Glasgow Coma Scale (GCS) was administered to all patients to assess initial neurological conditions. Computed tomography (CT) was utilized to determine the characteristics of the hematoma, including its nature, unilateral or bilateral hemispheric localization, maximum width on both sides in axial images, midline shift, and the presence of the radiological membrane.

The study excluded patients who exhibited a small size of hematoma (<1 cm), lacked neurological deterioration, or declined surgical intervention for various reasons. Additionally, patients who were ineligible for glucocorticoid therapy, individuals who were immunocompromised, those treated for infection or cancer, and those under anti-inflammatory therapy were also not enrolled.

Our study exclusively enrolled patients who underwent surgical intervention for a chronic subdural hematoma. The surgical procedure was scheduled for the patients after determining the location. A single burr-hole, double burr-hole, or mini-craniotomy has been performed as per the surgeon’s discretion during the surgical procedure. A subdural drain was inserted in all patients as a clinical, surgical principle. The magnitude of the thickest section of the hematoma was determined by analyzing the control CT scans conducted during the initial 24-hour period following the early postoperative phase and the second week following the surgery. The dimensions of the hematoma in the thickest section of the axial images were documented. Patients in the dexamethasone group received a randomized administration of 8 mg (4 mg twice a day) for two weeks and were subsequently monitored. Dexamethasone treatment was gradually discontinued at the end of the second week. In this process, the side effects that may occur in the patients were examined, and the complete blood count (CBC), C-reactive protein (CRP), and liver and kidney function tests in the second week were examined. The study documented the postoperative Glasgow Coma Scale (GCS) scores, hospitalization duration, and subdural drain placement following the surgical procedure.

Statistical analysis

Data were analyzed using the Statistics Package for the Social Sciences version 23.0 (IBM SPSS Statistics, Armonk, NY, USA). When applicable, nominal variables were reported as percentages and compared using a two-tailed Chi-square or Fisher’s test. Continuous quantitative variables were reported as mean ± standard deviation (SD) if they followed a Gaussian distribution; otherwise, they were represented as median (minimum-maximum). Parametric methods were used for measurement values suitable for normal distribution. In accordance with parametric methods, the independent sample t-test was used to compare the measurement values of two independent groups. Nonparametric methods were used for measurement values unsuitable for normal distribution. In accordance with nonparametric methods, the Mann-Whitney U test was used to compare the measurement values of two independent groups. Repeated-measures analysis of variance (ANOVA) test was used to evaluate the difference between repeated measures between groups. The p-value was set at <0.05 for statistical significance.

## Results

The study comprised 66 participants, with 33.3% (n=22) of them utilizing dexamethasone and the remaining 66.6% (n=44) not utilizing dexamethasone. Table 1 displays the distribution of demographic and clinical characteristics based on the utilization of dexamethasone by the patients. The study population consisted of individuals whose ages spanned from 14 to 92 years, with a median age of 73 years. Twenty patients, constituting 30.3% of the sample, were female, while 46 patients, accounting for 69.7% of the sample, were male. The study found that there was no significant difference in demographic and clinical characteristics between the two groups based on the treatments administered (p>0.05). The study findings indicate that a significant proportion (37%) of the patients who underwent surgery required reoperation. Notably, the rate of reoperation was found to be substantially lower (18%) among patients who received dexamethasone compared to those who did not receive the medication (47%). Although no instances of mortality were detected among the patients who received dexamethasone, four patients who did not receive the medication experienced mortality.

**Table 1:** Distribution of demographic and clinical findings

Characteristics	Total (N=66)	Dexamethasone (no) (n=44)	Dexamethasone (yes) (n=22)	p-value
	Number (%) or median (minimum-maximum)	Number (%) or median (minimum-maximum)	Number (%) or median (minimum-maximum)	
Age (years)	73 (14-92)	70 (14-90)	75 (38-92)	0.276
Female	20 (30.3)	16 (36.4)	4 (18.2)	0.218
Male	46 (69.7)	28 (63.6)	18 (81.8)	
Hematoma				1.000
Subacute	38 (57.6)	25 (56.8)	13 (59.1)	
Chronic	28 (42.4)	19 (43.2)	9 (40.9)	
Side				0.274
Bilateral	17 (25.8)	9 (20.5)	8 (36.4)	
Unilateral	49 (74.2)	35 (79.5)	14 (63.6)	
Midline shift (mm)	5.7 (0-16.7)	5.6 (0-16.7)	5.9 (0-14.1)	0.928
Right-side surgery				0.287
Double burr-hole	18 (50)	13 (54.2)	5 (41.7)	
Mini-craniotomy	6 (16.7)	5 (20.8)	1 (8.3)	
Single burr-hole	12 (33.3)	6 (25)	6 (50)	
Left-side surgery				0.494
Double burr-hole	22 (46.8)	15 (51.7)	7 (38.9)	
Mini-craniotomy	9 (19.1)	6 (20.7)	3 (16.7)	
Single burr-hole	16 (34)	8 (27.6)	8 (44.4)	
Neurological deficits	23 (34.8)	12 (27.3)	11 (50)	0.121
Subdural drain	65 (98.5)	43 (97.7)	22 (100)	1.000
Markwalder				0.265
0	10 (15.4)	7 (15.9)	3 (14.3)	
1	29 (44.6)	21 (47.7)	8 (38.1)	
2	22 (33.8)	12 (27.3)	10 (47.6)	
3	4 (6.2)	4 (9.1)	0 (0)	

Table 2 presents the outcomes of the ANOVA test conducted on multiple measurements to assess the variation in subdural dimensions before and after surgery, specifically during the second week post-operation, based on the administration of dexamethasone to the patients. Upon examination of the table, no significant difference was observed after the operation. A statistically significant difference was observed in the second-week post-surgery according to the utilization of treatment (p<0.043).

**Table 2:** Evaluation of the difference in measurements between groups SD: standard deviation

Subdural size (mm)	Treatment	Mean±SD	p-value
Early postoperative	Dexamethasone (no)	11.5±4.6	0.316
	Dexamethasone (yes)	12.7±4.1	
Second week postoperative	Dexamethasone (no)	9.2±4.7	0.043
	Dexamethasone (yes)	7.3±2.8	

**Figure 1:**
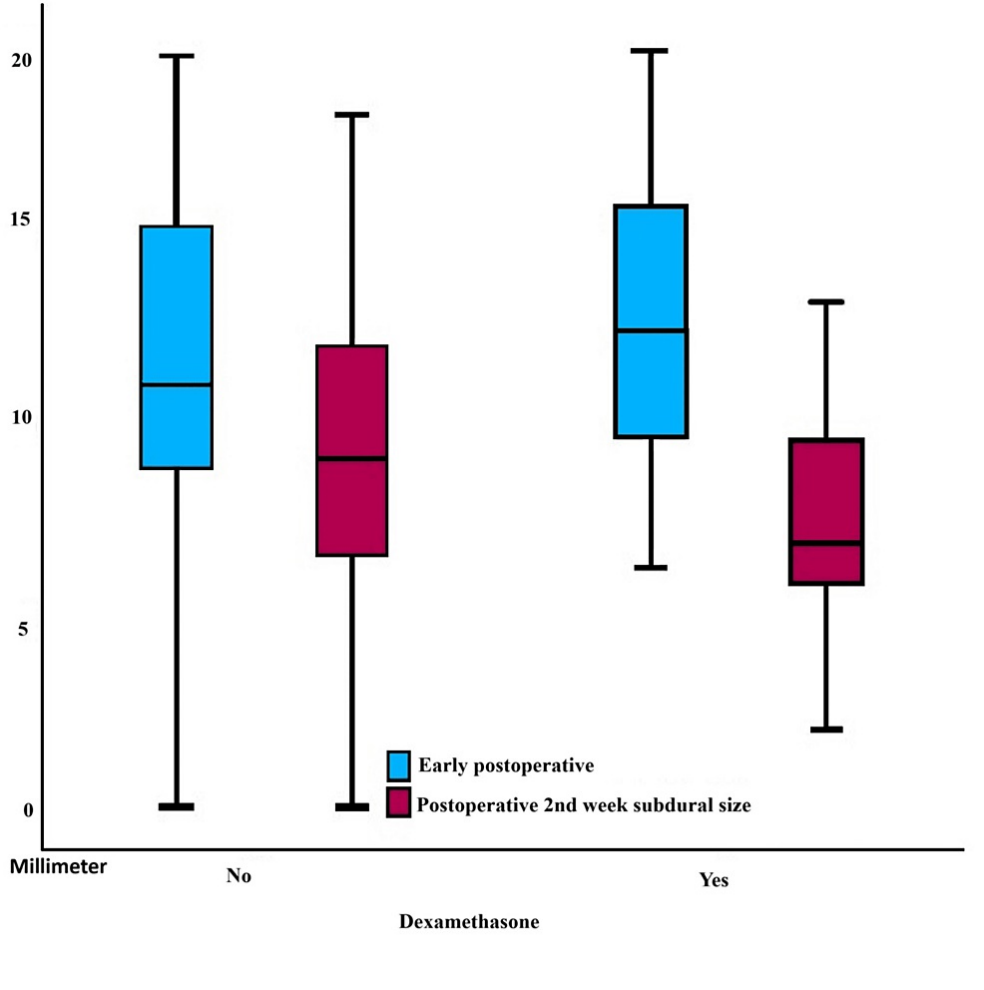
Comparison between early postoperative subdural size and the subdural size in the second week for patients who received dexamethasone and those who did not

No statistically significant difference was seen in the subdural dimensions of the patients after the initial surgical procedure. Upon analyzing the subdural dimensions of patients during the second week, a notable difference was detected between individuals who administered dexamethasone and those who did not (Figure 1). The efficacy of dexamethasone in subdural hematomas (SDHs) during the second week was established (p<0.001).

**Figure 2:**
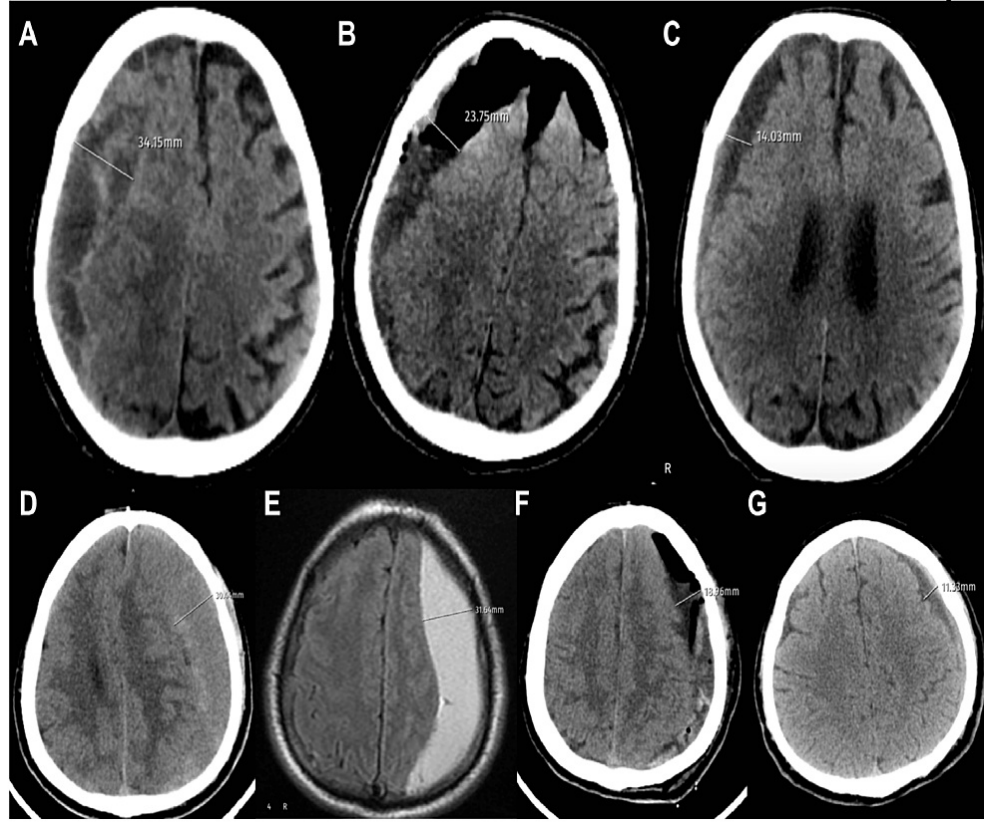
Examples of measurement of subdural hematoma sizes Right chronic subdural hematoma containing septa: A. preoperative CT image, B. early postoperative CT image, C. postoperative second-week image, D. preoperative CT image of chronic subdural hematoma in subacute formation on the left side, E. preoperative MRI image, F. early postoperative CT image, and G. postoperative second-week CT image. CT: computed tomography, MRI: magnetic resonance imaging

During the examination of subdural dimensions after the operation and in the second postoperative week, the preoperative radiological membrane, hematoma type, and trauma time were analyzed with respect to dexamethasone use (Figure 2). As a result, a difference has been detected between the radiological membrane and subdural dimensions. Still, there was no difference between the radiological membrane and subdural dimensions according to the treatment use. No statistically significant effect of hematoma type and trauma time has been observed in terms of subdural dimensions according to treatment use (p<0.05).

The result of evaluating whether there was a difference in the radiological membrane, hematoma type, and preoperative subdural size according to the presence of anticoagulation in the patients, the history of trauma, and the time of trauma are elaborated in Table 3. When the table is examined, a statistically significant correlation was found between anticoagulation and the type of hematoma (p<0.025) (Table 3).

**Table 3:** Differences in the radiological membrane, hematoma type, and preoperative subdural size SD: standard deviation

Variables	Anticoagulation			Trauma history			Trauma time	
	No	Yes		No	Yes		Median (minimum-maximum)	
	Number (%) or mean±SD	Number (%) or mean±SD		Number (%) or mean±SD	Number (%) or mean±SD			
Radiological membrane								
No	21 (50)	8 (33.3)	0.292	15 (45.5)	14 (42.4)	1.000	30 (3-210)	0.656
Yes	21 (50)	16 (66.7)		18 (54.5)	16 (57.6)		30 (7-60)	
Hematoma								
Subacute	29 (69)	9 (37.5)	0.025	17 (51.5)	9 (63.6)	0.455	30 (3-150)	0.909
Chronic	13 (31)	15 (62.5)		16 (48.5)	15 (36.4)		30 (10-210)	
Preoperative size (mm)	21.4±6.8	24.4±5.5	0.072	22.8±6.1	22.2±6.9	0.713		

According to the trauma history, there was no difference in the radiological membrane, hematoma type, and preoperative subdural size (p>0.05). Similarly, there was no statistically significant relationship between trauma time according to the radiological membrane and hematoma type and the type of surgery (p>0.05).

When we grouped the patients according to unilateral and bilateral surgical procedures, the results of the evaluation of the radiological membrane, hematoma type and duration of the drain, and the change in subdural size from preoperative to postoperative period are presented in Table 4. The study findings indicated that no significant statistical difference was observed in the reduction of subdural size with respect to the radiological membrane and hematoma type in both unilateral and bilateral operations (p>0.05) (Table 4).

**Table 4:** Results of the evaluation of other factors from the preoperative to the postoperative period SD: standard deviation

Side	Variables	Subdural size difference (preoperative-postoperative)	p-value
Unilateral	Radiological membrane, mean±SD		
	No	10.3±6.5	0.901
	Yes	10.5±4.3	
	Hematoma, mean±SD		
	Subacute	10.9±5.8	0.432
	Chronic	9.7±4.7	
	Drain duration	r=0.309	0.036
Bilateral	Radiological membrane, mean±SD		
	No	10.5±4.8	0.564
	Yes	11.6±2.8	
	Hematoma, mean±SD		
	Subacute	10.5±3.5	0.518
	Chronic	11.7±4.0	
	Drain duration	r=0.000	1.000

It was observed that there was a low linear positive correlation between the duration of the drain and the change in subdural size in those who had unilateral surgery. At the same time, there was no relationship between the duration of the resistance and the change in the subdural size in those who had bilateral operations.

The administration of dexamethasone did not result in any drug-related adverse effects among the patients. Furthermore, no further pathological observations were made in the blood tests conducted at the conclusion of the second week.

## Discussion

CSH arises from the rupture and hemorrhage of veins subsequent to physical injury [8]. Venous hemorrhage has a gradual progression and typically ceases through the application of intracranial pressure or the formation of a clot. The onset of symptoms in a subdural hematoma is comparatively gradual [9]. Typically, hemorrhaging in individuals who are youthful and in good health is observed in conjunction with considerably impactful physical injury, such as a collision involving a motorized vehicle. By way of contrast, elderly individuals may encounter instances of bleeding subsequent to minor physical trauma, such as a fall from a chair or a stumble on a carpeted surface. Subdural hematoma is a medically significant condition [10]. Failure to address intracranial pressure resulting from bleeding may lead to respiratory complications, paralysis, and fatality. Consequently, it is imperative to regard any impact to the cranial region as a significant occurrence [11].

Subdural hematoma (SDH) is bleeding between the dura and the arachnoid membrane caused by the bridging veins’ rupture from the brain surface to the dural sinuses. Sometimes, it can also be caused by arterial rupture in 20%-30% of cases. Approximately 11% of patients with subdural hematomas (SDHs) have mild to moderate traumatic brain injury [12]. Due to traumatic brain injury, the blood-brain barrier (BBB) is destroyed, and brain edema and intracranial pressure increase, which acts as a buffer for SDH. Most cases of SDH resulting from low-pressure venous bleeding stop due to increased intracranial pressure and hematoma [13]. In some cases, spontaneous SDH may occur, especially in the elderly. Anticoagulant and antiplatelet drug use, aneurysm rupture, vascular meningioma, arteriovenous malformation, dural metastasis, and intracranial hypotension can be listed among the causes of spontaneous SDH. It should be remembered that there may be SDH in head traumas, and the first preferred imaging method should be computed tomography (CT) [14-16].

Since the brain volume decreases in elderly patients, it does not pressure the brain vastly, and neurological disorders are not observed unless the hematoma volume becomes large [17]. Since the leakages in ruptures and ruptures of the bridging veins will fill the area within days, patients generally present with decreased hematodensity (due to the precipitation of the hematoma) in the subacute period. The literature has reported that the mean age is 64, and 80% of the cases are over 50 [18].

The most common complication after surgical treatment in patients with SH is a recurrence of bleeding. It has been reported that the most important reason for this situation is the incomplete resection of the membrane and the lack of expansion of the atrophic brain [19]. Another important problem is that rapid evacuation of the hematoma during surgery may cause a sudden decrease in intracranial pressure, resulting in brain shifts and acute hemorrhages in the opposite hemisphere [20]. Weigel et al. reported in their study that recurrence rates were 11%, 12%, and 18% for craniotomy, burr-hole drainage, and twist-drill craniotomy, respectively, and morbidity rates were 12%, 3.8%, and 3%, respectively [21].

The concomitant administration of dexamethasone and atorvastatin has been found to be more efficacious in mitigating inflammation and enhancing angiogenic activities. This combined therapy also promotes the stability of the local function and structure of the hematoma cavity, reduces the volume of hematomas, and yields better outcomes in rats with chronic subdural hematomas compared to monotherapy [22].

The administration of preoperative antiaggregant and oral anticoagulant treatments to patients, comprising 21% and 36% of the sample respectively, did not have any discernible impact on the profiles of inflammatory mediators, as per a study conducted on the subject [15]. The present study aimed to examine potential disparities in radiological membrane, hematoma type, and preoperative subdural size based on the presence of anticoagulation. We found a statistically significant correlation between anticoagulation and the type of hematoma (p<0.025).

Preoperatively, dexamethasone reduces the concentrations of inflammatory mediators intraoperatively and postoperatively. Most patients had dexamethasone preoperatively for 1-3 days and had a rapid-onset, reaching peak plasma concentration within 1-1.5 hours with a biological half-life of 36-54 hours. It may take time for the anti-inflammatory effect to seep into the subdural collection. Postoperative drain samples showed an overall reduction in the increase in many of the most inflammatory markers with dexamethasone compared to placebo, with an expected anti-inflammatory response. This is related to dexamethasone targeting inflammatory markers produced in response to surgery acutely after surgery. At the same time, the large preexisting pool was drained. A significant change was observed for vascular endothelial growth factor (VEGF) on day 2, which no longer showed postoperative peak concentrations [12]. Still, despite the small numbers, there is an indication that the anti-inflammatory role of dexamethasone allows the postoperative inflammatory response to be “damped,” especially VEGF. This may help explain the mechanism behind the role of dexamethasone in reducing the recurrence of chronic subdural hematoma (CSH) (from 7.1% to 1.7%) found in a large randomized trial with favorable side effects in these patients [23]. The present study aimed to examine potential disparities in subdural dimensions between the postoperative period and the second week following the operation, depending upon the administration of dexamethasone to patients. A statistically significant difference was observed in the second week post-operation with respect to treatment utilization (p<0.043). Furthermore, two of the patients who passed away in our investigation expired due to the emergence of acute subdural hematoma after two days of reoperation. One patient experienced the development of pulmonary embolism as a complication of anesthesia. One patient expired due to septic shock caused by an infection. The outcome demonstrates the potential hazards and repercussions of performing repeat surgical procedures on geriatric patients. This result validates the previously mentioned infiltration of dexamethasone during the postoperative phase.

According to the systematic review conducted by Berghauser Pont et al., it was observed that dexamethasone could potentially be beneficial in treating chronic subdural hematoma (CSH) based on the findings of five observational studies, which utilized either monotherapy or in combination with surgical intervention [24]. Following monotherapy, a substantial proportion of patients (ranging from 83% to 97%) exhibited favorable neurological outcomes. In contrast, the administration of supplementary dexamethasone was associated with good outcomes in a higher percentage of patients (ranging from 87% to 100%), whereas surgical intervention resulted in good outcomes in a comparatively lower proportion of patients (ranging from 64% to 92%). Another study proposed that extended preoperative administration of corticosteroids was linked to a decreased recurrence rate in the surgical management of chronic subdural hematoma (CSH) using burr-hole craniotomy [25]. According to another study, the administration of postoperative corticosteroids has been shown to be efficacious in mitigating disease recurrence among patients [26-28]. The administration of dexamethasone resulted in a reduction of subdural dimensions during the postoperative period, specifically in the second week, as observed in our study. The study can be planned with a larger number of patients and prospectively. The limitation of this study is that it was partially done with a small number of patients.

## Conclusions

Regarding the outcomes of this research, we can conclude that dexamethasone was not associated with any early adverse events. Additionally, dexamethasone could leverage reoperation prevention for the elderly with various comorbidities.
